# Factors explaining variation in recommended care pathways following hospital-presenting self-harm: a multilevel national registry study

**DOI:** 10.1192/bjo.2020.116

**Published:** 2020-11-25

**Authors:** Eve Griffin, David Gunnell, Paul Corcoran

**Affiliations:** School of Public Health, University College Cork, Ireland; and National Suicide Research Foundation, Ireland; National Institute for Health Research Bristol, Biomedical Research Centre, University Hospitals Bristol and Weston NHS Foundation Trust and University of Bristol, UK; and Population Health Sciences, University of Bristol, UK; National Suicide Research Foundation, Ireland

**Keywords:** Self-harm, outpatient treatment, emergency services, emergency psychiatry, hospital services

## Abstract

**Background:**

People who present to hospital following self-harm are at high risk of suicide. Despite this, there are considerable variations in the management of this group across hospitals and the factors influencing such variations are not well understood.

**Aims:**

The aim of this study was to identify the specific hospital and individual factors associated with care pathways following hospital-presenting self-harm.

**Method:**

Data on presentations to hospitals by those aged 18 years and over were obtained from the National Self-Harm Registry Ireland for 2017 and 2018. Factors associated with four common outcomes following self-harm (self-discharge, medical and psychiatric admission and psychosocial assessment before discharge) were examined using multilevel Poisson regression models.

**Results:**

Care pathways following self-harm varied across hospitals and were influenced by both hospital and individual factors. Individual factors were primarily associated with self-discharge (including male gender, younger age and alcohol involvement), medical admission (older age, drug overdose as a sole method and ambulance presentations) and psychiatric admission (male gender, methods associated with greater lethality and older age). The hospital admission rate for self-harm was the only factor associated with all outcomes examined. The availability of psychiatric in-patient facilities and specialist mental health staff contributed to variation in psychiatric admissions and psychosocial assessments prior to discharge. Hospital factors explained the majority of observed variation in the provision of psychosocial assessments.

**Conclusions:**

Characteristics of the presenting hospital and hospital admission rates influence the recommended care pathways following self-harm. Provision of onsite mental health facilities and specialist mental health staff has a strong impact on psychiatric care of these patients**.**

Patients who present to hospital as a result of self-harm are one of the groups at highest risk of suicide.^1^ National clinical guidelines and quality standards for the management of self-harm in adults outline standard aspects of short-term care, including the provision of a comprehensive assessment of an individual's psychosocial needs and mental health status.^2,3^ However, evidence for improved patient outcomes following routine aspects of clinical management is mixed. A systematic review found little evidence that routine aspects of hospital management reduce risk of subsequent self-harm or suicide,^4^ whereas more recent studies have shown that the provision of a psychosocial assessment is associated with reduced rates of repetition.^5,6^ One reason for a lack of consistency in findings may be the degree of variation in the care of self-harm across hospitals in rates of psychosocial assessment and admissions.^7–10^ Such inconsistencies in the quality of care received following self-harm can have a significant negative impact for individuals, particularly those who leave before their care has been completed.^11^ Patient factors associated with aspects of care, including psychosocial assessment and self-discharge, have been previously studied, with factors such as degree of medical severity, age, time of presentation and self-harm history all influencing clinical management.^12–16^ Little is known about the specific hospital factors that influence how such presentations are managed, however, they are likely to be related to resources, structure of service provision and availability of specialist mental health staff.^7,17^

In this study we investigated the recommended care pathways following hospital-presenting self-harm, using a multilevel approach. Data from a national self-harm registry was used to examine the individual- and hospital-specific factors associated with four common clinical outcomes – self-discharge, medical admission, psychiatric admission and provision of a psychosocial assessment prior to discharge from the emergency department.

## Method

### Study design

The study was conducted using data from the National Self-Harm Registry Ireland, covering the period 1 January 2017 to 31 December 2018. The Registry is a national monitoring system of self-harm attendances to hospital emergency departments in Ireland and data are gathered by trained data registration officers. The definition of self-harm used by the Registry is ‘an act with non-fatal outcome in which an individual deliberately initiates a non-habitual behaviour, that without intervention from others will cause self-harm, or deliberately ingests a substance in excess of the prescribed or generally recognised therapeutic dosage, and which is aimed at realising changes that the person desires via the actual or expected physical consequences’.^18^

We based our analysis on patients aged 18 years and over, presenting to the 26 acute general hospitals that provide a 24 h emergency department service. Within the Irish healthcare system, these hospitals fall under seven hospital groups, each with their own governance structure. The groups are constructed according to geographical locations, as well as combining hospitals varying in model status, size and speciality. We excluded presentations made to the emergency department of paediatric hospitals (*n* = 3) as well as presentations made to hospitals reconfigured as local injury units (*n* = 13), as these hospitals had their emergency departments closed or were operating with reduced hours during the study period.

### Ethics statement

Ethical approval for the National Self-Harm Registry Ireland has been granted by the National Research Ethics Committee of the Faculty of Public Health Medicine. The Registry operates a waiver of consent, granted by the Health Research Consent Declaration Committee.

### Individual-level data

As it is difficult to ensure that an individual's first presentation to hospital in the study time period was their first ever presentation, the analysis was restricted to individuals who did not attend hospital with self-harm in the 3 years preceding the study period (i.e. between 1 January 2014 and 31 December 2016). This cohort represented 14 555 (70.2%) of all presentations recorded during the study period 1 January 2017 to 31 December 2018. This was done to maximise the number of true first presentations and to accurately examine the impact of recent self-harm history, by controlling the analyses for the total number of presentations made by an individual during the study period.

Data collected for each self-harm presentation included the following: age, gender and area of residence; medical card status (whether the individual had access to free medical services, based on income and/or health status); time and day of presentation; method(s) of self-harm; whether alcohol was consumed as part of the self-harm act; and whether the patient was brought to hospital via ambulance services (a marker of the severity of the presentation). Methods of self-harm were coded according to the World Health Organization's ICD-10 for intentional injury (X60-84).^19^ Drug overdose involved the intentional ingestion of medications and illegal substances (X60–64). Drug names and the quantity of tablets ingested in overdoses, involving up to 13 drugs, are captured in the Registry via hospital medical records and, where present, toxicology reports. Attempted hanging (X70), attempted drowning (X71) and self-cutting (X78) were also examined individually. Other, less common methods of self-harm, were grouped into a category of ‘other methods’ (X66–69; X72–77; X79–84). Alcohol involvement was coded by X65.

The Pobal HP Deprivation Index was used as a measure of socioeconomic deprivation. This deprivation measures consists of three dimensions – demographic profile, social class composition and labour market situation.^20^ There are 3409 small areas in Ireland known as electoral divisions. For each self-harm presentation, a deprivation score was assigned according to the individual's area of residence. Areas were divided into quintiles based on their absolute deprivation scores.

### Hospital-level data

For the purposes of the multilevel analyses, data were also gathered at the level of the hospital to which self-harm presentations were made. This included routinely available information as well as information about mental health service resources at each hospital site during the study period.

Several hospital characteristics were investigated in the analyses. These were: availability of a clinical nurse specialist for self-harm;^21^ psychiatric in-patient facilities;^22^ hospital location (city or town); and the type of hospital (general or tertiary)^23^. The following variables were also included: the number of all emergency department attendances per year;^22^ number of in-patient beds per 1000 emergency department attendances;^24^ and the number of patients on trolleys in the emergency department per 1000 emergency department attendances^25^ (a marker for both overcrowding in the emergency department and a lack of in-patient beds in the hospital).

Given the wide variation in admission rates across hospitals, the hospital admission rate – the conversion rate from emergency department attendance to emergency admission – was constructed for all emergency presentations^26^ and separately for self-harm presentations. This measure has been shown to reflect underlying hospital policies on management of attendances as well as a lack of specialist services in hospitals.^27^

All continuous hospital-level measures were transformed into categorical data with two equal sized categories, using the Stata *xtile* command. Given the wider range of self-harm admission rate values (8.2–53.0%), three equal sized categories were used for this variable. Prior to transformation, the intercorrelations of these measures were assessed, with no strong associations (*r* greater than +/−0.50) observed. A full list and description of these data sources and the distribution of hospital measures by hospital are presented in Supplementary Tables 1 and 2 available at https://doi.org/10.1192/bjo.2020.116.

### Outcome measures

Information regarding the care pathways following each presentation included whether a patient was given a psychosocial assessment in the emergency department, whether medical or psychiatric in-patient admission was recommended, and if the patient self-discharged. It was also recorded if a patient was admitted to an emergency department observation unit (also known as medical assessment unit, clinical decision unit or accident and emergency ward) in the emergency department during their hospital visit.

The four outcomes considered in this study were as follows.

- Self-discharge: the Registry records if an individual left the emergency department without being triaged (registered only) or before a next-care recommendation could be made. Some aspects of medical care may have been received (for example wound treatment or pain management) and an initial psychosocial assessment may have been conducted. However, a final decision on the recommended next care was not completed by hospital staff in these instances. In a minority of these presentations a psychosocial assessment was recorded as having been conducted (*n* = 183, 10.4%).
- Medical admission: medical admission was recorded if the patient was admitted into a medical ward of the presenting hospital, but not if the patient was transferred to another hospital. As the Registry records the care that is recommended by the presenting hospital, this category also includes a small number of presentations where medical admission was recommended, but the individual refused (*n* = 96; 0.7%).
- Psychiatric admission: psychiatric admission was selected if the patient was admitted to a psychiatric ward in a facility of the hospital (onsite or offsite). Presentations that were transferred to another hospital for psychiatric admission are not recorded by the Registry.
- Psychosocial assessment: as outlined by the National Institute for Health and Care Excellence guidelines,^2^ every patient attending an emergency department following self-harm should receive a preliminary psychosocial assessment at the time of attendance. This assessment should ‘determine a person's mental capacity, their willingness to remain for further (psychosocial) assessment, their level of distress and the possible presence of mental illness’. It is recorded that a psychosocial assessment was conducted if the assessment occurred in the emergency department at the time of attendance, or if an assessment was offered but the patient refused. This assessment is conducted by a specialist mental health professional. It is possible that patients admitted to the presenting hospital may have received an assessment at a later stage, but this is not recorded by the Registry. For this reason, analysis of this outcome was limited to those who were discharged from the emergency department following treatment.

### Statistical analyses

A multilevel Poisson regression model was constructed to establish the factors contributing to each of the four outcomes examined. The Poisson model, with robust standard errors, was preferred over a logistic model, given that most of the outcomes were common, and in order to generate incidence rate ratios (IRRs) and their 95% confidence intervals.^28^ As there was limited variation at the level of hospital group, this was excluded in the formal analyses. The multilevel models were constructed using the *xtmepoisson* command (mixed effects Poisson model) in Stata.

Hospital was included as a random effect, with specific hospital-level and individual-level factors entered into the models as fixed effects. Variables were entered into the models in a backward method, beginning with hospital-level data. Variables that were not strongly associated (*P* < 0.05) with the outcome measure were removed sequentially, until models with variables significantly associated with the outcome remained. Given the number of variables considered in the hierarchical models, a sensitivity analysis was undertaken, in order to assess the robustness of the results to different approaches. A forward stepwise regression analysis was conducted, where the first variable added to the model was the one that gave the greatest increase in the log likelihood. Further variables were added based on their effect on the log likelihood, as long as the effect was statistically significant. These models were constructed separately for the hospital- and individual-level factors, before entering the selected variables into the multilevel model. Both approaches identified the same set of variables in the final multivariable models.

In order to calculate the intraclass correlation coefficient (ICC) – a measure of how much of the total variance is attributable to clustering within hospitals – the command *xtmelogit* was used (mixed-effects logistic model), as there is no equivalent option for *xtmepoisson* in Stata. Funnel plots were used to illustrate variation across hospital sites, plotting the four outcomes against the number of self-harm presentations to each hospital, using exact binomial 95% and 99.8% control limits to account for overdispersion.^29^ All analyses were conducted using Stata/IC 12.0.

## Results

### Participant characteristics

Between 1 January 2017 and 31 December 2018, there were a total of 14 555 hospital presentations that were a result of self-harm by those aged 18 years and over. Of these, 7317 (50.3%) were by women and the mean age was 32 years (interquartile range 23–45). The majority of presentations involved drug overdose only (*n* = 8264, 56.8%). Of these, the most common drug types recorded were analgesics (*n* = 3397; 41.1%) and minor tranquillisers (*n* = 2974; 35.7%). Almost one-fifth of presentations involved self-cutting only (*n* = 2645, 18.2%). A minority of presentations involved both a drug overdose and self-cutting (*n* = 647; 4.4%). Attempted hanging and attempted drowning were recorded as the method of self-harm in 6.1% (*n* = 885) and 2.6% (*n* = 384) of presentations, respectively. The remaining presentations (*n* = 1730, 11.9%) involved less common methods of self-harm, including self-poisoning, jumping from a height or before a moving object and firearm discharge. Alcohol was involved in 34.0% (*n* = 4943) of presentations. The 14 555 presentations were made by 11 971 individuals, implying that 17.8% of presentations (*n* = 2584) were due to repeat self-harm.

**Fig. 1:**
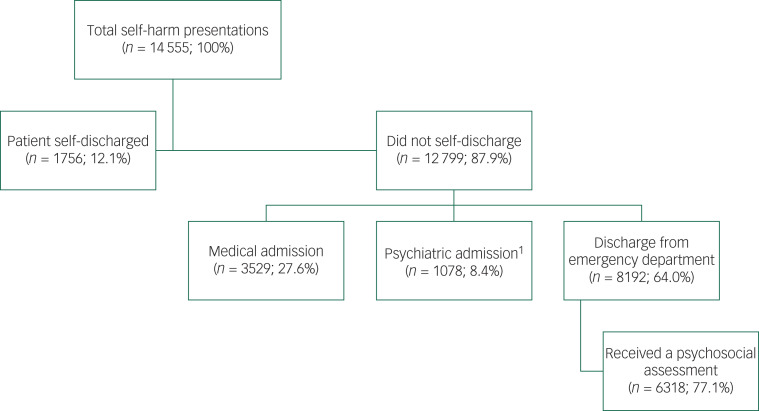
Care pathways following hospital-presenting self-harm ^1^It may not always be recorded if a patient was admitted to an offsite psychiatric facility. In addition those which were transferred to another hospital for psychiatric admission are not included here, so this figure is likely to be an underestimate.

Figure 1 illustrates the care pathways following presentation to the emergency department. In total, 12.1% (*n* = 1756) of presentations resulted in the patient leaving the emergency department before a next-care recommendation could be made (self-discharge). Of the remaining presentations, more than one-third (*n* = 4607; 36.0%) were admitted into the presenting hospital. For more than a quarter (*n* = 3529; 27.6%) the individual was admitted to a medical ward, with 1078 (8.4%) admitted to a psychiatric ward. Of these admissions, it was recorded that an assessment was conducted in the emergency department before admission in 82.5% (*n* = 3609) of these admissions. The majority of presentations not resulting in self-discharge were subsequently discharged from the emergency department following treatment (*n* = 8192; 64.0%), with 6318 (77.1%) of these receiving a psychosocial assessment prior to discharge.

### Variation by hospital

There was significant variation in the recommended care pathways following self-harm by individual hospital. The proportion of presentations resulting in self-discharge ranged from 4.7% to 17.8%. Rates of medical and psychiatric admission ranged from 8.2% to 53.0% and 0.3% to 28.3%, respectively. Rates of discharge from the emergency department ranged from 27.5% to 72.2%, and of those, the provision of psychosocial assessments ranged from 17.4% to 97.1%.

**Fig. 2:**
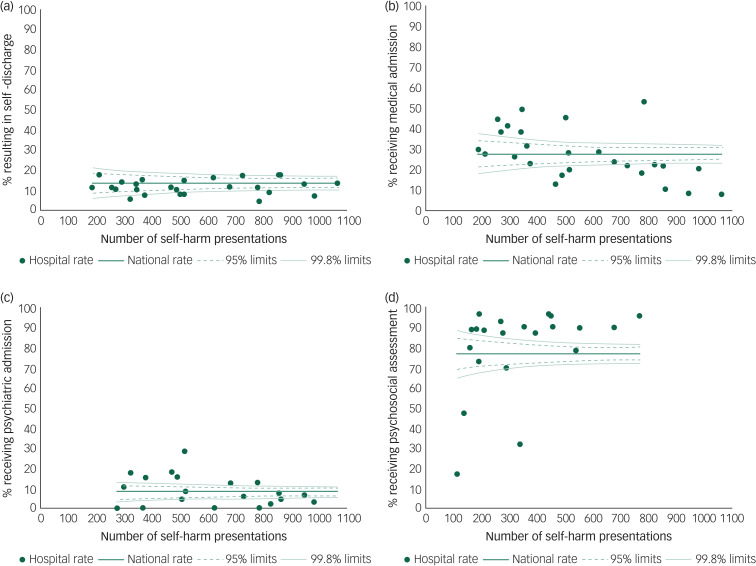
Funnel plots demonstrating the variation by hospital in the proportion of presentations resulting in (a) self-discharge, (b) medical admission, (c) psychiatric admission or (d) psychosocial assessment prior to discharge.

The plots presented in Fig. 2(a–d) demonstrate this variation across hospital sites, according to the number of presentations made. For a large number of hospitals, their values lie outside the confidence intervals, indicating that their outcomes are not within the expected range. This is most pronounced for medical admission, where 16 hospitals lie outside the expected range, and for psychosocial assessment prior to discharge, where 17 hospitals lie outside the expected range.

### Factors contributing to recommended care pathways following self-harm

The results of the multilevel regression models are presented in Table 1. The univariable associations between each hospital and individual factor with the four outcomes examined, adjusted for variation at a hospital level, are provided in Supplementary Tables 3 and 4.

**Table 1:** Adjusted multilevel Poisson regression models with hospital-level and individual-level factors associated with recommended care pathways following self-harm

	Self-discharge	Medical admission^a^	Psychiatric admission^a^	Psychosocial assessment^b^
	IRR (95% CI)	IRR (95% CI)	IRR (95% CI)	IRR (95% CI)
*Hospital-level factors*				
Hospital admission rate for self-harm (ref, low <0.19)				
Medium (0.19–0.26)	0.54 (0.38–0.77)	1.71 (1.26–2.33)	1.49 (0.48–4.66)	1.10 (0.79–1.53)
High (>0.26)	0.76 (0.56–1.02)	2.87 (2.18–3.78)	0.31 (0.10–0.91)	0.69 (0.50–0.96)
Hospital location (ref, Dublin City)				
Other city	0.53 (0.35–0.80)	–	–	–
Town	0.66 (0.44–0.97)	–	–	–
Psychiatric in-patient facilities onsite (ref, offsite)	–	–	2.78 (0.99–7.76)	1.75 (1.03–2.96)
Dedicated self-harm clinical nurse specialist (ref, no)	–	–	5.77 (1.24–26.88)	–
*Individual-level factors*				
Male gender (ref, female)	1.30 (1.18–1.43)	–	1.16 (1.03–1.32)	–
Age group (ref, ≥55 years)				
<30 years	1.54 (1.27–1.88)	0.60 (0.54–0.67)	0.60 (0.49–0.74)	–
30–54 years	1.67 (1.37–2.02)	0.77 (0.70–0.84)	0.90 (0.74–1.09)	–
Method of self-harm (ref, drug overdose only)				
Self-cutting only	1.28 (1.14–1.44)	0.28 (0.24–0.32)	1.38 (1.16–1.65)	–
Drug overdose and self-cutting	0.70 (0.55–0.90)	0.65 (0.53–0.78)	0.92 (0.63–1.35)	–
Attempted hanging only	0.52 (0.40–0.68)	0.39 (0.32–0.48)	2.67 (2.20–3.23)	–
Attempted drowning only	0.71 (0.50–0.99)	0.44 (0.33–0.59)	2.09 (1.54–2.84)	–
Other methods	0.71 (0.60–0.85)	0.60 (0.53–0.68)	2.11 (1.78–2.51)	–
Alcohol involved (ref, no)	1.38 (1.25–1.53)	–	0.67 (0.57–0.78)	–
Brought by ambulance (ref, other mode of arrival)	1.20 (1.08–1.32)	1.39 (1.29–1.49)	0.77 (0.68–0.88)	–
Presented outside 9.00 to 17.00 h (ref, no)	1.53 (1.36–1.71)	0.89 (0.83–0.95)	0.85 (0.74–0.96)	–
Medical card holder (ref, no)	–	–	0.94 (0.91–0.98)	–
Previous self-harm presentations within study period (ref, no)	1.21 (1.08–1.35)	–	1.25 (1.08–1.45)	–
Admitted to emergency department observation unit (ref, no)	–	0.78 (0.69–0.88)	0.57 (0.42–0.76)	1.17 (1.08–1.27)

IRR, incidence rate ratio; ref, reference group.

a. Also adjusted for psychosocial assessment.

b. Limited to presentations where the individual was discharged from the emergency department following treatment.

A combination of individual- and hospital-specific factors explained the variation in the likelihood of self-discharge. At a hospital level, presentations to hospitals with higher admission rates for self-harm presentations were associated with a reduced risk of self-discharge, as were presentations made to hospitals located outside of Dublin City. At an individual-level, the following factors increased risk of self-discharge: male gender, younger age, self-cutting as the sole method of self-harm, alcohol involvement and previous self-harm presentations. In addition, presentations made out of normal working hours and those brought to hospital by ambulance were more likely to self-discharge. On the other hand, presentations involving both drug overdose and self-cutting, as well as methods associated with higher potential lethality (including attempted hanging and drowning) were less likely to result in self-discharge.

The factors associated with medical admission were largely at the individual level. Not surprisingly, at a hospital level, presentations made to hospitals with higher admission rates for self-harm was the only factor to be positively associated with medical admission. Considering individual-level factors, risk of medical admission varied according to the method of self-harm, and was lower for presentations involving methods other than drug overdose. Those aged less than 55 years, those who had consumed alcohol and presented outside normal working hours were less likely to receive medical admission, as were those who were initially admitted to an emergency department observation unit. Those brought to hospital by ambulance were also more likely to be medically admitted.

Considering admission to a psychiatric ward, several individual and hospital factors contributed to the observed variation. Presentations made to hospitals with onsite psychiatric in-patient facilities were more likely to be admitted to a psychiatric ward than those where the facilities were located offsite, as were presentations made to hospitals with a dedicated self-harm clinical nurse specialist. On the other hand, presentations made to hospitals with high admission rates for self-harm were less likely to be admitted to a psychiatric ward. At an individual level, the likelihood of psychiatric admission was increased where it was a repeat self-harm presentation. Compared with presentations involving a drug overdose only, self-cutting, attempted hanging, attempted drowning and other methods of self-harm were associated with a higher risk of psychiatric admission. A lower risk of psychiatric admission was associated with younger age (particularly those aged under 30 years). The following factors also reduced risk of psychiatric admission: alcohol involvement, arrival at hospital via ambulance, presenting outside normal working hours, patients initially admitted to an emergency department observation unit, along with medical card holders.

Hospital factors accounted for the majority of variation observed in the provision of a psychosocial assessment prior to discharge from the emergency department. Presentations made to hospitals with psychiatric in-patient facilities onsite were more likely to receive a psychosocial assessment. Conversely, presentations to hospitals with high self-harm admission rates were less likely to receive a psychosocial assessment. At an individual level, patients admitted to an emergency department observation unit prior to discharge were more likely to receive a psychosocial assessment.

Table 2 details the observed variance at a hospital level across the four outcomes examined. The ICCs from the null model (including hospital only as a random effect) ranged from 0.12 to 0.44. This signals the proportion of total variance in the outcome variable at the hospital level, with an ICC value of 0.44, for example, indicating that 44% of the observed variance is at the hospital level. After adjustment for both individual and hospital covariates, there remains a degree of unexplained variation between hospitals. This variation was lowest when considering medical admission and moderate for the other outcomes. The final models containing hospital and individual fixed factors demonstrated a marked reduction in unexplained variance, suggesting that there is little residual variance from unobserved factors (ICC less than 20% for three outcomes). This remaining variance was highest for psychosocial assessments, at 26%. However, in this model the variance reduced from 39% initially, suggesting that the model has accounted for a large part of the variance, but not all.

**Table 2:** Intraclass correlation coefficients (ICC) and 95% confidence intervals

	ICC (null)	ICC (adjusted)	Percentage of hospital variance explained by the model
Self-discharge	0.39 (0.27–0.53)	0.02 (0.01–0.04)	94.9
Medical admission	0.12 (0.07–0.19)	0.10 (0.06–0.17)	16.7
Psychiatric admission	0.44 (0.28–0.61)	0.21 (0.12–0.34)	52.3
Psychosocial assessment	0.39 (0.27–0.53)	0.26 (0.16–0.39)	33.3

## Discussion

We have demonstrated that the recommended care pathways for individuals who self-harm vary considerably by hospital and are influenced by a range of factors, both at a hospital and individual level. In particular, hospital factors such as the self-harm admission rate and availability of in-patient psychiatric facilities onsite contribute significantly to the observed variation in the management of self-harm across hospitals. Although the hospital location also contributed to risk of self-discharge, socioeconomic deprivation was not associated with any of the outcomes examined.

The factors at an individual level that were associated with admission to a medical or psychiatric ward reflect the need for such admissions. Older people, presentations involving drug overdose and self-cutting, and those arriving via ambulance services were more likely to receive medical admission. Psychiatric admission was more common among those who had a history of previous self-harm, and where the method of self-harm involved attempted hanging, reflecting the higher risk of suicide among these patients.^30^ Individual-level factors identified as increasing risk of self-discharge are similar to those identified in previous studies.^12,13^ The range of associated factors identified – including male gender, self-cutting as a method, alcohol involvement, presenting out-of-hours and to hospitals located in Dublin City – reflecting the complexity of such attendances, and suggest that there may be specific challenges with regards to clinical management.

Unlike previous studies that have reported a range of patient characteristics associated with not receiving a psychosocial assessment (including younger age, presenting out-of-hours, repeat self-harm, self-cutting as a method, socioeconomic deprivation),^12,31–33^ we found that hospital factors explained almost all of the variation in this aspect of clinical care, indicating that receiving a psychosocial assessment is dependent on adequate resources in hospitals, particularly the availability of onsite psychiatric facilities, rather than based on perceived suicidal risk of an individual patient.

To account for the fact that self-harm presentations are made to largely heterogeneous emergency departments, we included several hospital measures reflecting both mental health resources and the profile of the hospital more generally. In the final multivariable models, general hospital factors did not make a significant contribution. The admission rate of self-harm presentations was associated with all outcomes examined. This rate (also known as a conversion rate) of a hospital emergency department is often used as a measure of turnover in the context of examining potential avoidable admissions^34^ and in this study, may reflect underlying admission policies across hospitals for this patient group.^21^ This type of resource utilisation has previously been reported as being an important contributor to appropriate care following self-harm, with integrated care pathways and short-term medical admission resulting in increased psychosocial assessments, decreased medical complications, shorter length of stay and cost savings.^17,35^

Future research could consider the reasons underlying hospital admission policies, for example if an individual is admitted because of a psychosocial assessment not being possible at the time of presentation, or where a decision regarding in-patient admission or transfer to another hospital for specialist care cannot be made, because of limited resources at the time of presentation. This may explain the lower rate of psychiatric admissions in hospitals with high conversion rates. Hospitals may also have a preference to admit individuals who present with self-harm, as well as utilising emergency department observation units, in order to offset the demands of meeting service completion targets. A recent study on meeting waiting time targets in the UK suggests that adherence to service targets such as completing treatment for self-harm patients within 4 hours is associated with more restrictive measures and may have a negative influence on quality of care.^36^ Mental health triage systems, which have been shown to be effective in improving responses to self-harm, along with appropriate use of emergency department observation units, were also suggested as a mechanism to help offset the pressures of completing self-harm care within 4 hours, particularly for complex cases. This would be reflected in our data, whereby the use of emergency department observation units^37^ may reduce the need for subsequent admission and allow for a psychosocial assessment to be conducted at an appropriate time.

Psychiatric in-patient facilities being present onsite and the availability of a dedicated clinical nurse specialist for self-harm were significant factors in determining psychiatric admission, as would be expected. Onsite psychiatric in-patient facilities also influenced the provision of psychosocial assessments prior to discharge. This may be due to mental health staff being more available to conduct assessments in hospitals with psychiatric in-patient facilities. The availability of liaison psychiatry services has been shown to be associated with improved care for self-harm in acute settings,^38,39^ and having dedicated staff with training may help to ensure positive patient experiences and future help-seeking, as well as providing appropriate responses and care pathways. In 2015, a national clinical programme was implemented to standardise services for self-harm in acute settings in Ireland, providing funding for dedicated self-harm nurses to work in each emergency department. The components of the programme include the provision of a psychosocial assessment, appropriate referrals with secondary care, and involving families and support as appropriate.^27^ In this study the associations with increased psychosocial assessments and lower risk of self-discharge observed at a univariable level (Supplementary Table 3) held but did not remain significant in the final multivariable models. The latter may be because of a lack of power, given that only two hospitals were operating without such a staff member during the study period. The impact of such resource allocation should be considered in greater detail in future studies, including the out-of-hours availability of such staff. We did not have information on the availability of psychiatric services by time of day, nor did we have complete data on waiting times for emergency department attendances. However, given that we demonstrated an increased risk of self-discharge and reduced admissions when patients presented outside of normal working hours, the availability of 24/7 services should be reviewed as standard measures across hospitals.

### Strengths and limitations

There are several strengths to this study. We used data from a national self-harm registry, covering all acute hospitals in Ireland. We also gathered accurate data on hospital factors from a wide range of sources, which correspond to the study time period. We used multilevel modelling techniques to account for variation across hospitals (random effects) as well as fixed hospital and individual factors. This approach is commonly used in health services research, but has not been comprehensively applied to examine quality of care for self-harm in emergency settings. The ICC statistics generated from the full models were lower than for the null models, indicating that a large proportion of observed variation was accounted for in our models. This small amount of residual variance may be explained by data on other factors that were not available to us. Importantly, we did not have complete individual-level data. Clinical data related to precipitants of self-harm, current and previous psychiatric diagnoses, as well as recent or ongoing engagement with mental health services were not available, all of which could have an impact on the care pathways received in hospital. In addition, we did not have accurate information on the degree of medical severity of the self-harm presentations examined.

At a hospital level, more detailed data on staffing and protocols with regards the management of mental health-related presentations were not available. We also did not have detailed information on specific aspects relating to the quality of care received, such as the format of the psychosocial assessments provided, whether they included a comprehensive needs assessment, or if they were conducted by a mental health professional. Similarly, we did not have information on length of stay for patients who were admitted. Future research examining the quality of specific aspects of clinical management should incorporate such information.

### Implications

Immediate and effective care for those who present to hospital with self-harm is imperative, given the risk of repeat self-harm in the short term and of suicide in the medium term.^40^ The hospital costs of self-harm are significant^41^ and investment in such measures may result in savings with regards to admissions and repeat presentations. Mental health-related presentations represent a small but high-risk proportion of all hospital presentations, and should be considered in the allocation of hospital resources. Appropriate management of self-harm presentations to hospital requires a coordinated response to both the psychological and physical needs of the individuals. Our findings demonstrate that the variation of care pathways following self-harm across hospitals is largely driven by the resources and policies existing in the individual hospitals. The integration of mental health services in acute settings – including onsite psychiatric facilities and dedicated mental health staff – improves psychiatric care, indicating that the further integration of acute mental health protocols and resources is warranted.

## Data Availability

Access to data from the National Self-Harm Registry Ireland may be requested by contacting the National Suicide Research Foundation (info@nsrf.ie).

## Supplementary material

For supplementary material accompanying this paper visit http://dx.doi.org/10.1192/bjo.2020.116. click here to view supplementary material
