# Supplementary material for: Factors explaining variation in recommended care pathways following hospital-presenting self-harm: a multilevel national registry study
Source: BJPsych Open. 2020 Nov 25;6(6):e145. doi: 10.1192/bjo.2020.116 (PMC7745229; doi:10.1192/bjo.2020.116)
Supplement: Supplementary file 1 [file S2056472420001167sup001.docx]

Table 1. Description of hospital-level variables

| Measure | Construction of measure |
| --- | --- |
| Availability of clinical nurse specialist (self-harm) | Dedicated self-harm nurse specialist in hospital (yes/no)^22^ |
| Psychiatric inpatient facilities | Psychiatric inpatient facilities available onsite or offsite^23^ |
| All emergency department attendances^23^ | Number per year |
| Number of inpatient beds^24^ per 1,000 emergency department attendances | Based on number of inpatient medical beds in hospital |
| Number of emergency department trolleys^25^ per 1,000 emergency department attendances | Based on monthly average number of trolleys recorded in emergency department |
| Hospital admission rate (self-harm) | Ratio of all self-harm attendances by the number of self-harm attendances medically admitted |
| Hospital admission rate (all presentations) | Ratio of all emergency department attendances by the number of emergency admissions^26^ |
| Type of hospital | General hospital (Model 3 – providing 24/7 acute surgery, acute medicine and critical care) or Tertiary hospital (Model 4 – with additional supra regional or national specialist centres)^23^ |
| Hospital location | Hospital situated in Dublin City, Other City, or town |

Note: All data relate to 2018

Supplementary Table 2. Distribution of hospital factors by hospital.

|  | Number of hospitals, 26 (100%) |
| --- | --- |
| Admission rate (self-harm) |  |
| Less than 0.19 (low) | 7 (26.9%) |
| Between 0.19 and 0.26 (medium) | 7 (26.9%) |
| Greater than 0.26 (high) | 12 (36.2%) |
| Admission rate (all) |  |
| Less than 0.26 (low) | 14 (53.8%) |
| Greater than 0.26 (high) | 12 (46.2%) |
| Hospital location |  |
| Dublin City | 6 (23.1%) |
| Other City | 5 (19.2%) |
| Town | 15 (57.7%) |
| Number of inpatient beds per 1,000 emergency attendances |  |
| Less than 7.2 per 1,000 (low) | 16 (61.5%) |
| Greater than 7.2 per 1,000 (high) | 19 (73.1%) |
| Number of trolleys per 1,000 emergency attendances |  |
| Less than 4.7 per 1,000 (low) | 15 (57.7%) |
| Greater than 4.7 per 1,000 (high) | 11 (42.3%) |
| Hospital Type |  |
| General hospital (Model 3) | 17 (65.4%) |
| Tertiary hospital (Model 4) | 9 (34.6%) |
| Dedicated self-harm Clinical Nurse Specialist |  |
| Yes | 24 (92.3%) |
| No | 2 (7.7%) |
| Psychiatric inpatient facilities |  |
| Onsite | 17 (65.4%) |
| Offsite | 9 (34.6%) |

Supplementary Table 3. Poisson regression models demonstrating the univariable associations between hospital-level factors and clinical management of self-harm.

|  | **Self-discharge** | **Medical admission** | **Psychiatric admission** | **Psychosocial assessment^1^** |
| --- | --- | --- | --- | --- |
|  | IRR (95% CI) | IRR (95% CI) | IRR (95% CI) | IRR (95% CI) |
| Hospital admission rate for self-harm (ref=low, <0.16) |  |  |  |  |
| Medium (0.19-0.26) | 0.81 (0.57-1.14) | 1.77 (1.38-2.27) | 0.78 (0.20-3.04) | 0.98 (0.67-1.42) |
| High (>0.26) | 0.85 (0.62-1.16) | 2.92 (2.33-3.65) | 0.13 (0.03-0.45) | 0.58 (0.42-0.82) |
| Hospital admission rate for all (ref=low, <0.26) | 0.82 (0.64-1.07) | 1.07 (0.73-1.57) | 2.04 (0.58-7.21) | 1.26 (0.90-1.77) |
| Hospital type (ref= Model 3) | 0.99 (0.75-1.30) | 0.68 (0.47-0.99) | 1.57 (0.41-5.94) | 1.25 (0.88-1.78) |
| Number of trolleys per 1,000 emergency attendances (ref=low, <4.7) | 1.04 (0.79-1.36) | 0.79 (0.54-1.16) | 0.74 (0.20-2.66) | 0.85 (0.60-1.21) |
| Number of inpatient beds per 1,000 emergency attendances (ref=low, <7.2) | 0.87 (0.66-1.14) | 0.98 (0.66-1.46) | 2.44 (0.68-8.76) | 1.20 (0.85-1.71) |
| Hospital location (ref=Dublin City) |  |  |  |  |
| Other City | 0.78 (0.53-1.14) | 0.85 (0.51-1.40) | 1.03 (0.16-6.82) | 0.88 (0.53-1.45) |
| Town | 0.87 (0.64-1.19) | 1.58 (1.06-2.36) | 0.48 (0.10-2.25) | 0.70 (0.47-1.04) |
| Psychiatric inpatient facilities onsite (ref=offsite) | 0.94 (0.71-1.25) | 0.63 (0.44-0.91) | 5.20 (1.48-18.28) | 1.38 (0.98-1.93) |
| Dedicated self-harm Clinical Nurse Specialist (ref=no) | 0.79 (0.53-1.18) | 1.03 (0.56-1.88) | 2.43 (0.32-18.63) | 1.35 (0.79-2.33) |

Note: Univariable associations adjusted for clustering at hospital level

^1^Limited to presentations where the individual was discharged from the emergency department following treatment

IRR=Incidence rate ration; CI = Confidence Interval

Supplementary Table 4. Poisson regression models demonstrating the univariable associations between individual-level factors and clinical management of self-harm.

|  | **Self-discharge** | **Medical admission** | **Psychiatric admission** | **Psychosocial assessment^1^** |
| --- | --- | --- | --- | --- |
|  | IRR (95% CI) | IRR (95% CI) | IRR (95% CI) | IRR (95% CI) |
| Male gender (ref=female) | 1.29 (1.18-1.42) | 0.85 (0.79-0.91) | 1.32 (1.17-1.49) | 0.99 (0.94-1.04) |
| Age group (ref=55 years+) |  |  |  |  |
| <30 years | 1.55 (1.28-1.88) | 0.47 (0.43-0.52) | 0.69 (0.57-0.84) | 0.99 (0.90-1.08) |
| 30-54 years | 1.74 (1.44-2.10) | 0.68 (0.62-0.75) | 0.89 (0.74-1.08) | 0.99 (0.90-1.08) |
| Method of self-harm (ref=drug overdose only) |  |  |  |  |
| Self-cutting only | 1.28 (1.14-1.44) | 0.23 (0.20-0.27) | 1.53 (1.29-1.81) | 0.98 (0.92-1.04) |
| Drug overdose and self-cutting | 0.77 (0.60-0.99) | 0.64 (0.53-0.76) | 0.99 (0.68-1.44) | 1.03 (0.92-1.15) |
| Attempted hanging only | 0.53 (0.41-0.69) | 0.37 (0.30-0.45) | 3.49 (2.91-4.18) | 1.05 (0.95-1.16) |
| Attempted drowning only | 0.78 (0.56-1.10) | 0.43 (0.33-0.57) | 2.21 (1.64-2.99) | 1.03 (0.90-1.19) |
| Other methods | 0.73 (0.62-0.86) | 0.55 (0.49-0.62) | 2.56 (2.17-3.02) | 0.99 (0.92-1.08) |
| Alcohol involved (ref=no) | 1.51 (1.37-1.66) | 1.04 (0.97-1.12) | 0.56 (0.48-0.65) | 0.99 (0.95-1.05) |
| Brought by ambulance (ref=other mode of arrival) | 1.29 (1.17-1.42) | 1.63 (1.51-1.75) | 0.62 (0.55-0.70) | 0.99 (0.95-1.05) |
| Presented outside 9am-5pm (ref=no) | 1.70 (1.52-1.90) | 0.87 (0.81-0.93) | 0.71 (0.63-0.80) | 0.98 (0.93-1.03) |
| Presented at weekend (ref=weekday) | 1.12 (1.01-1.24) | 1.02 (0.95-1.10) | 0.89 (0.78-1.01) | 0.96 (0.91-1.01) |
| Medical card holder (ref=no) | 0.98 (0.95-1.01) | 0.97 (0.95-0.99) | 0.93 (0.90-0.97) | 1.00 (0.98-1.02) |
| Previous self-harm presentations within study period (ref=no) | 1.27 (1.13-1.42) | 0.87 (0.79-0.95) | 1.30 (1.12-1.50) | 1.01 (0.94-1.07) |
| Admitted to emergency department observation unit (ref=no) | 0.98 (0.94-1.01) | 0.86 (0.83-0.89) | 0.92 (0.87-0.99) | 0.98 (0.95-1.01) |
| Deprivation quintile (ref=least deprived) |  |  |  |  |
| 2 | 0.88 (0.75-1.04) | 1.03 (0.93-1.15) | 0.94 (0.78-1.13) | 0.99 (0.92-1.08) |
| 3 | 0.92 (0.79-1.07) | 0.99 (0.88-1.10) | 1.00 (0.83-1.21) | 0.98 (0.90-1.07) |
| 4 | 1.01 (0.86-1.17) | 1.02 (0.91-1.14) | 0.98 (0.80-1.19) | 0.99 (0.91-1.07) |
| Most deprived | 0.95 (0.81-1.11) | 1.01 (0.90-1.14) | 0.81 (0.65-1.02) | 0.98 (0.90-1.06) |

Note: Univariable associations adjusted for clustering at hospital level

^1^Limited to presentations where the individual was discharged from the emergency department following treatment

IRR=Incidence rate ration; CI = Confidence Interval
